# Combined Hepatocellular Neuroendocrine Carcinoma: A Rare Tumor

**DOI:** 10.14309/crj.0000000000000931

**Published:** 2023-07-08

**Authors:** Zunirah Ahmed, Mukul K. Divatia, Suzanne Crumley, David W. Victor, Sudha Kodali

**Affiliations:** 1 Division of Gastroenterology and Hepatology, Houston Methodist, Houston, TX; 2 Department of Pathology and Genomic Medicine, Houston Methodist, Houston, TX; 3 Sherrie and Alan Conover Center for Liver Disease and Transplantation, Houston Methodist Hospital, TX

**Keywords:** hepatocellular carincoma, neuroendocrine tumor, rare tumor

## Abstract

Neuroendocrine tumors originate from neuroendocrine cells primarily located in the gastrointestinal tract. These tumors often metastasize to the liver. Primary hepatic neuroendocrine carcinomas are uncommon, and combined hepatocellular neuroendocrine carcinomas are exceedingly rare. There is a lack of data on the management of these rare tumors. Most cases have very poor prognosis secondary to aggressive behavior of the neuroendocrine tumor component. It is important for clinicians to be aware of this rare carcinoma to allow for early diagnosis and optimize potential treatment options.

## INTRODUCTION

Hepatocellular carcinoma (HCC) is the seventh most commonly diagnosed neoplasm worldwide.^1^ Excellent cross-sectional imaging modalities have become standard of care in diagnosing HCCs, and histological diagnosis for HCC is no longer required in most patients. However, HCC has a wide morphological spectrum, and it can contain additional population of malignant cells, such as cholangiocarcinoma, neuroendocrine, and sarcomatous components.^2^ Combined HCC-neuroendocrine carcinoma (NEC) tumors are histologically categorized into 2 subtypes: small and large cell types.^3,4^ The collision type tumor is characterized by 2 synchronized, but histologically distinct tumors, which are derived from the same organ with no histologic admixture, whereas the combined-type tumor includes intermingled components which cannot be separated in the transitional area within a single tumor nodule.^5^ We present a challenging case of mixed HCC-NEC, highlighting the importance of biopsy for lesions with atypical features for HCCs.

## CASE REPORT

**Figure 1.**
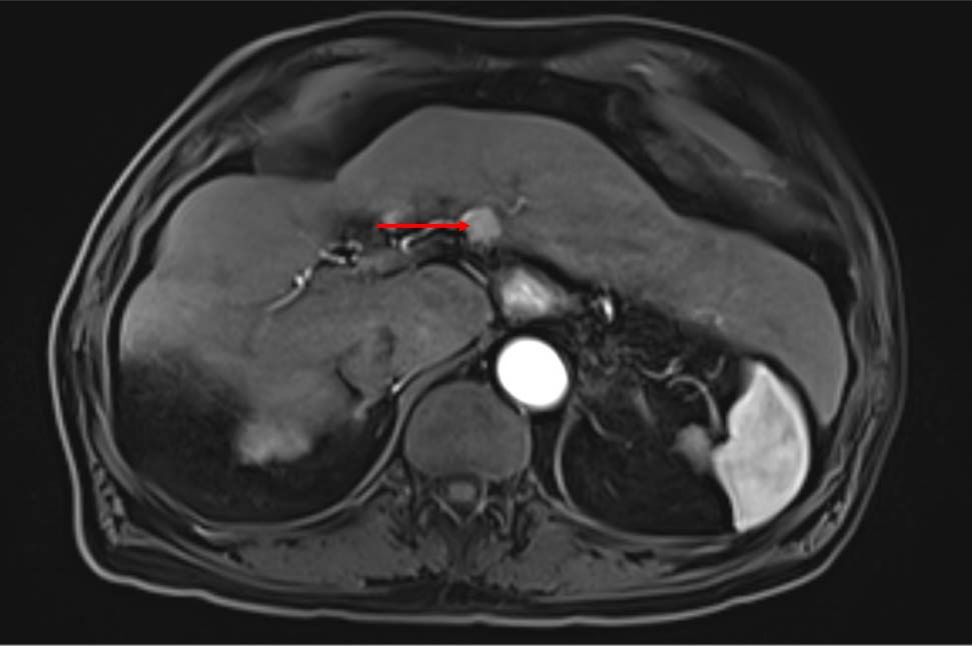
Abdominal magnetic resonance imaging with a 1.6 × 1.4 cm arterially enhancing lesion in the segment 3.

**Figure 2.**
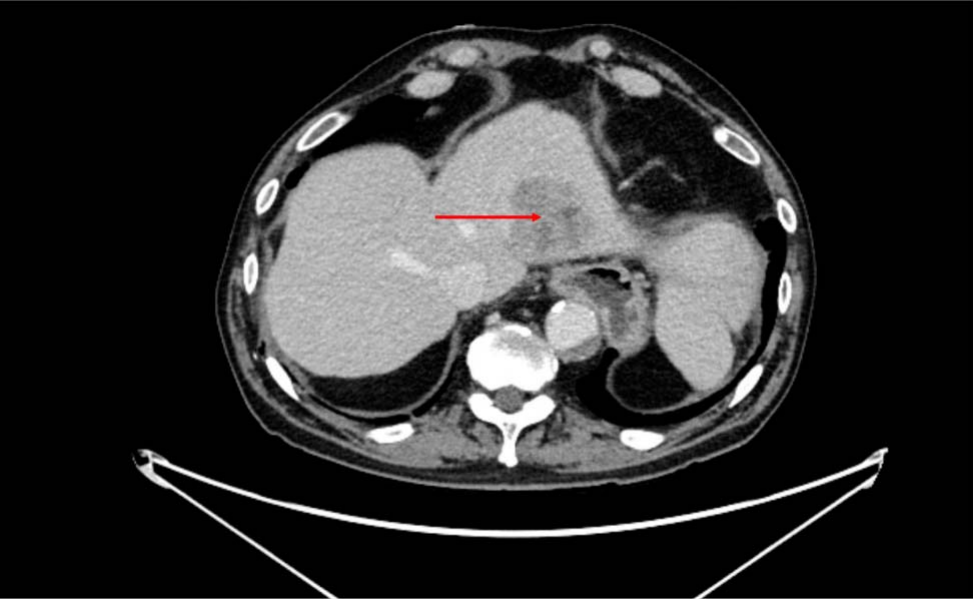
Abdominal computed tomography with contrast with a 5.0 × 4.7 cm lesion.

A 67-year-old man presented with a medical history significant for esophageal cancer status post esophagectomy, cirrhosis due to nonalcoholic steatohepatitis, and biopsy-proven hepatocellular carcinoma status post transarterial chemoembolization and radiofrequency ablation. Although the patient was listed for liver transplantation, he unfortunately had hemorrhagic stroke and coronary artery disease, debilities that led to him getting delisted. Through this period, he continued to have imaging and had no evidence of tumor recurrence. Abdominal magnetic resonance imaging showed a 1.4 × 1.6 cm arterially enhancing Liver Imaging Reporting and Data System-5 lesion in hepatic segment 3 (Figure 1) on surveillance imaging 2 years after his radiofrequency ablation in the previously treated area. Laboratory data were unremarkable except for an alpha-fetoprotein (AFP) level of 158 from 8.4 ng/mL. Given the size of lesion, a 3-month follow-up imaging was recommended, which showed an increase in size to 5.0 × 4.7 cm (Figure 2). The AFP level was 179 ng/mL. Carcinoembryonic antigen and CA 19-9 were 4.3 and 18 ng/mL, respectively. The radiological appearance of lesion was not classic for HCC, and given concern for mixed tumor, an image-guided biopsy was recommended at our multidisciplinary tumor board conference.

**Figure 3.**
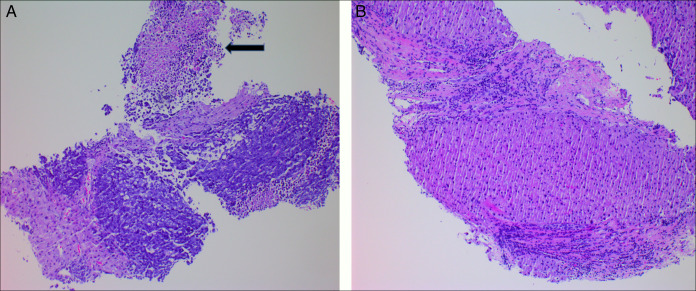
(A) Small cell neuroendocrine carcinoma with characteristic morphologic features and accompanying tumor necrosis (arrow) and (B) background hepatic parenchyma with nodular cirrhosis (A and B, hematoxylin and eosin stain, 100×).

**Figure 4.**
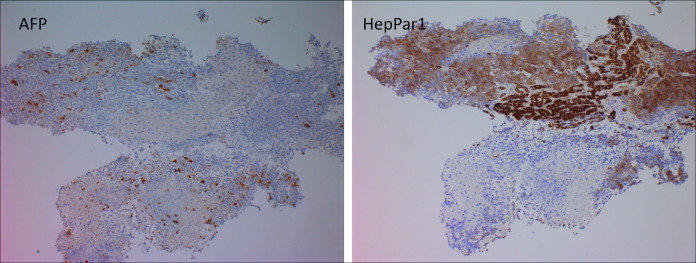
Focal expression of AFP and strong positivity for HepPar1 in tumor cells, supportive of the hepatic origin of small cell neuroendocrine carcinoma (immunoperoxidase, 100×). AFP, alpha-fetoprotein.

**Figure 5.**
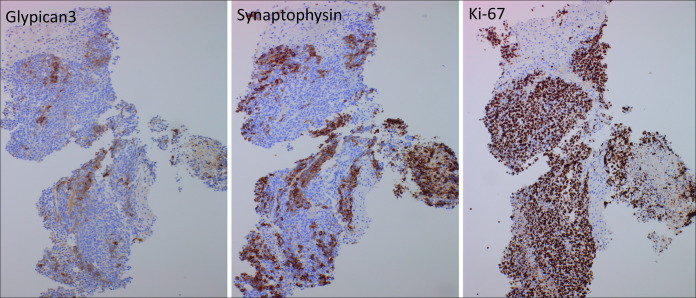
Focal glypican 3 staining with strong positivity for neuroendocrine differentiation marker synaptophysin and a markedly increased Ki-67 proliferation index (80%–90 %) in tumor cells. (immunoperoxidase, 100×).

Biopsy demonstrated features of a high-grade carcinoma with tumor cells positive for pancytokeratin cocktail, neuroendocrine differential marker synaptophysin (diffuse), and chromogranin (focal, paranuclear) and negative for cytokeratin 7, cytokeratin 20, PAX8, prostate specific antigen, NKX3.1, arginase, and CD45. Glypican 3 immunohistochemical showed patchy expression, whereas AFP and HepPar1 both showed positive staining in the tumor cells. The Ki67/MIB1 proliferation rate was 80%–90% in the tumor cells. In addition to these immunohistochemical findings, the morphologic features of this high-grade carcinoma were also consistent with those of small cell neuroendocrine carcinoma, including small-to-medium-sized cells with scant cytoplasm, nuclear hyperchromasia with molding, inconspicuous nucleoli, necrosis, and apoptosis. Therefore, a diagnosis of combined HCC-NEC was established in conjunction with the previously biopsied HCC. These aforementioned findings are shown in Figures 3–5.

**Figure 6.**
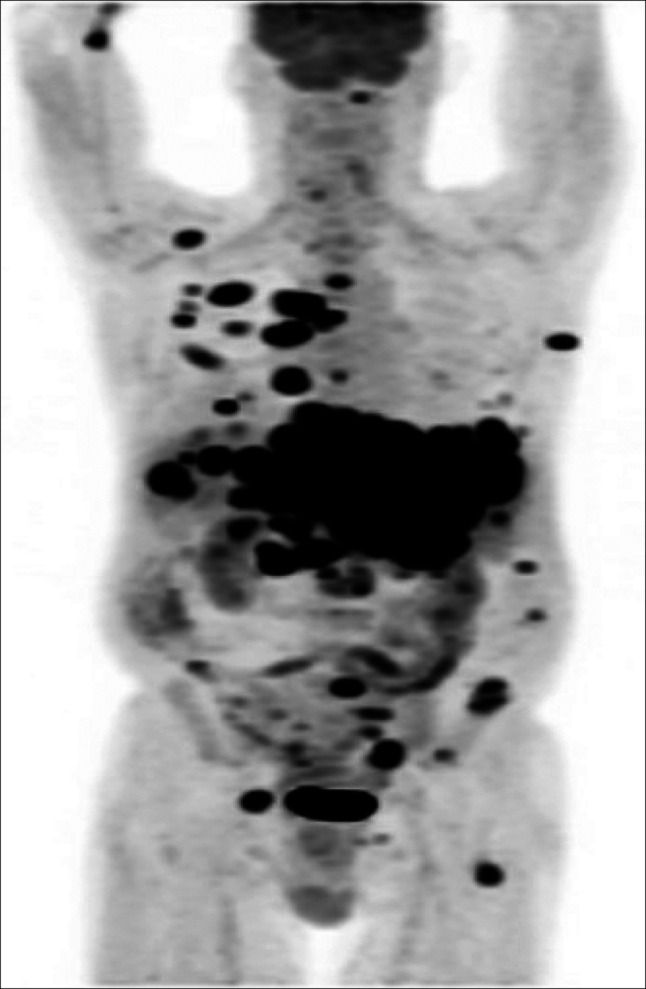
A positron emission tomography scan with widely metastatic lesions.

Oncology was consulted, and positron emission tomography–computed tomography of the skull to midthigh showed widespread metastatic disease with numerous lesions in the lungs, chest, and skeleton (Figure 6). This was striking because the chest and bone computed tomography scan 5 months earlier had demonstrated no metastatic disease. AFP increased to 4,977 ng/mL over the course of 1 month. Clinically, the patient declined rapidly with respiratory distress, hepatic encephalopathy, sepsis, and given the rapid deterioration, his family and him chose hospice care.

## DISCUSSION

Combined HCC-NEC are very rare tumors. Nomura et al investigated 1,235 tumors, and the incidence of HCC-NEC was found to be approximately 0.4%.^5^ The pathogenesis of mixed HCC-NEC remains unclear. There are 2 predominant hypotheses regarding the origin of these rare tumors, which includes differentiation of hepatic stem cells into both NEC and HCC components^6^ vs well or moderately differentiated HCCs undergoing neuroendocrine differentiation.^7^ Yamaguchi et al found that immunohistochemically, in combined tumors, some tumor cells of the HCC may be immunoreactive for neuroendocrine markers.^8^ In addition, the expression of p53 protein and Ki-67 proliferative index was found to be notably higher in the NEC component than in the HCC component.^8^

A recent review of all published cases of mixed HCC-NEC by Nakano et al showed that most of the cases were combined type, occurred in male patients, and hepatitis B and hepatitis C were the leading cause of chronic liver disease.^9^ This review also brought to attention that almost all the cases were diagnosed as HCC preoperatively and then rediagnosed after resection as HCC-NEC. Only 3 cases were diagnosed with biopsy, fine-needle aspiration, or autopsy.^9^ This and our case emphasize the fact that biopsy should be considered for diagnosis when the tumor pattern is not typical for HCC because radiological diagnosis can be inaccurate.

It is not correct to classify a carcinoma as combined HCC-NEC based only on immunohistochemical findings in a tumor demonstrating a monomorphic growth pattern, such as focal glypican-3 or HepPar1 immunostaining in a case of neuroendocrine carcinoma or focal staining for neuroendocrine markers such as synaptophysin or CD56 immunohistochemical staining in a conventional hepatocellular carcinoma. In our case, the NEC component also expressed AFP immunohistochemically, thus supporting its origin from a previously biopsied and proven conventional HCC.

These tumors can arise in both cirrhotic and noncirrhotic livers without any specific etiologic association. The proportion of both components in such tumors varies from one case to another; however, most of these tumors are primarily composed of the HCC component, whereas the metastatic lesions exhibit an NEC component.

Optimal assessment of prognosis for combined HCC-NEC is limited by the scant number of reported cases. However, studies in the published literature have documented that such tumors have aggressive behavior with a poor outcome similar to primary hepatic NEC rather than conventional HCC.^5,8,10,11^

## DISCLOSURES

Author contributions: Z. Ahmed wrote and revised the manuscript; M. Divatia and S. Crumley provided with the pathology images and revised the manuscript; and DW Victor and S. Kodali wrote and revised the manuscript and is the article guarantor.

Financial disclosure: None to report.

Informed consent was obtained for this case report.

## References

[1] McGlynn KA Petrick JL El‐Serag HB. Epidemiology of hepatocellular carcinoma. Hepatology. 2021;73(S1):4–13. 10.1002/hep.31288 PMC7577946 32319693

[2] Garcia MT Bejarano PA Yssa M Buitrago E Livingstone A. Tumor of the liver (hepatocellular and high grade neuroendocrine carcinoma): A case report and review of the literature. Virchows Archiv. 2006;449(3):376–81. 16896889 10.1007/s00428-006-0251-0

[3] Choi GH Ann SY Lee SI Kim SB Song IH. Collision tumor of hepatocellular carcinoma and neuroendocrine carcinoma involving the liver: Case report and review of the literature. World J Gastroenterol. 2016;22(41):9229. 27895410 10.3748/wjg.v22.i41.9229 PMC5107604

[4] Baker E Jacobs C Martinie J Iannitti DA Vrochides D Swan RZ. Mixed hepatocellular carcinoma, neuroendocrine carcinoma of the liver. Am Surg. 2016;82(11):1121–5. 28206942

[5] Nomura Y Nakashima O Akiba J . Clinicopathological features of neoplasms with neuroendocrine differentiation occurring in the liver. J Clin Pathol. 2017;70(7):563–70. 27881473 10.1136/jclinpath-2016-203941

[6] Beard RE Finkelstein SD Borhani AA Minervini MI Marsh JW. A massive hepatic tumor demonstrating hepatocellular, cholangiocarcinoma and neuroendocrine lineages: A case report and review of the literature. Int J Surg case Rep. 2017;37:26–32. 28623758 10.1016/j.ijscr.2017.05.039 PMC5475262

[7] Ishida M Seki K Tatsuzawa A . Primary hepatic neuroendocrine carcinoma coexisting with hepatocellular carcinoma in hepatitis C liver cirrhosis: Report of a case. Surg Today. 2003;33(3):214–8. 12658390 10.1007/s005950300048

[8] Yamaguchi R Nakashima O Ogata T Hanada K Kumabe T Kojiro M. Hepatocellular carcinoma with an unusual neuroendocrine component. Pathol Int. 2004;54(11):861–5. 15533230 10.1111/j.1440-1827.2004.01770.x

[9] Nakano A Hirabayashi K Yamamuro H . Combined primary hepatic neuroendocrine carcinoma and hepatocellular carcinoma: Case report and literature review. World J Surg Oncol. 2021;19(1):78. 33726764 10.1186/s12957-021-02187-5 PMC7968236

[10] Yang C-S Wen M-C Jan Y-J Wang J Wu C-C. Combined primary neuroendocrine carcinoma and hepatocellular carcinoma of the liver. J Chin Med Assoc. 2009;72(8):430–3. 19686999 10.1016/S1726-4901(09)70400-9

[11] Nakanishi C Sato K Ito Y . Combined hepatocellular carcinoma and neuroendocrine carcinoma with sarcomatous change of the liver after transarterial chemoembolization. Hepatol Res. 2012;42(11):1141–5. 23094854 10.1111/j.1872-034X.2012.01017.x

